# Large-scale omics dataset of polymer degradation provides robust interpretation for microbial niche and succession on different plastisphere

**DOI:** 10.1038/s43705-023-00275-z

**Published:** 2023-07-03

**Authors:** Daiki Yokoyama, Ayari Takamura, Yuuri Tsuboi, Jun Kikuchi

**Affiliations:** 1 RIKEN Center for Sustainable Resource Science, 1-7-22 Suehiro-cho, Tsurumi-ku, Yokohama, Kanagawa 230-0045 Japan; 2 Graduate School of Medical Life Science, Yokohama City University, 1-7-29 Suehiro-cho, Tsurumi-ku, Yokohama, Kanagawa 230-0045 Japan; 3 Graduate School of Bioagricultural Sciences, Nagoya University, 1 Furo-cho, Chikusa-ku, Nagoya, Aichi 464-0810 Japan

**Keywords:** Environmental chemistry, Microbiome

## Abstract

While biodegradable polymers have received increased attention due to the recent marine plastic problem, few studies have compared microbiomes and their degradation processes among biodegradable polymers. In this study, we set up prompt evaluation systems for polymer degradation, allowing us to collect 418 microbiome and 125 metabolome samples to clarify the microbiome and metabolome differences according to degradation progress and polymer material (polycaprolactone [PCL], polybutylene succinate-co-adipate [PBSA], polybutylene succinate [PBS], polybutylene adipate-co-terephthalate [PBAT], and poly(3-hydroxybutyrate-co-3-hydroxyhexanoate) [PHBH]). The microbial community compositions were converged to each polymer material, and the largest differences were observed between PHBH and other polymers. Such gaps were probably formed primarily by the presence of specific hydrolase genes (i.e., 3HB depolymerase, lipase, and cutinase) in the microorganisms. Time-series sampling suggested several steps for microbial succession: (1) initial microbes decrease abruptly after incubation starts; (2) microbes, including polymer degraders, increase soon after the start of incubation and show an intermediate peak; (3) microbes, including biofilm constructers, increase their abundance gradually. Metagenome prediction showed functional changes, where free-swimming microbes with flagella adhered stochastically onto the polymer, and certain microbes started to construct a biofilm. Our large-dataset-based results provide robust interpretations for biodegradable polymer degradation.

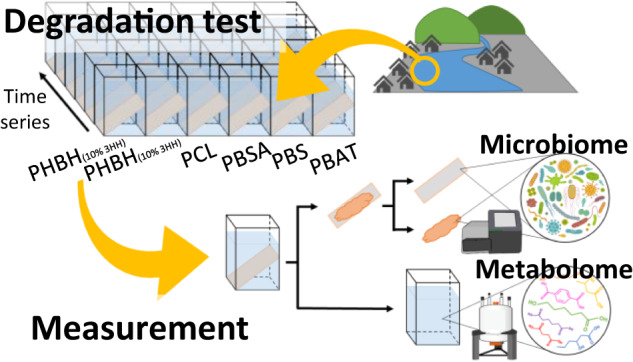

## Introduction

Plastics have become indispensable in our society and are ubiquitously present in diverse fields, including packaging, building materials, automotives, electronic devices, daily necessities, agriculture, etc. [1]. In 2019, global plastic production reached 368 million metric tons [1]. A large proportion of the plastics produced are wasted every year, with 4.8–12.7 million metric tons entering the ocean in 2010 [2]. These plastics are incorporated into the food web and nutrient cycling in marine ecosystems without being degraded and can potentially affect biological metabolisms [3].

This problem has led to increasing interest in the use of biodegradable polymers in place of conventional plastics. Biodegradable polymers, particularly marine-degradable plastics, are thought to avoid the formation of microplastics in the marine environment; therefore, marine-degradable plastics are acceptable under the Regulation, Evaluation, and Authorization of Chemicals regulations (REACH). The most widespread biodegradable polymer is polylactic acid (PLA); however, PLA decomposes extremely slowly in seawater [4]. Several polymers that are biodegradable in seawater have been developed; for example, polycaprolactone (PCL), polybutylene succinate-co-adipate (PBSA), polybutylene succinate (PBS), polybutylene adipate-co-terephthalate (PBAT), and poly(3-hydroxybutyrate-co-3-hydroxyhexanoate) (PHBH). Biodegradable polymers can be classified into two categories based on how they are synthesized: chemically and biologically synthesized polymers [5]. Biodegradable polymers produced by chemical synthesis include PCL, PBSA, PBS, and PBAT. PCL is an aliphatic polyester produced by the ring-opening polymerization of ε-caprolactone [6, 7]. PBSA, PBS, and PBAT are copolymers composed of 1,4-butanediol and dicarboxylic acids (succinate and adipate for PBSA, succinate for PBS, and adipate and terephthalate for PBAT) [7, 8]. Biologically synthesized polymers include PHBH, a copolymer of 3-hydroxybutyrate (3HB), and 3-hydroxyhexanoate (3HH). PHBH is a polyhydroxyalkanoate (PHA) that is produced by certain microbes in carbon-rich but nutrient-deficient environments for energy storage [6, 9].

These biodegradable polymers can be degraded by microbes in nature, which form a unique microbiome on their surface. This unique ecosystem is known as a “plastisphere” [10–12]. The first attack on the polymer structure involves enzymatic hydrolysis by microbe-produced hydrolases. Since hydrolases include various enzymes with different substrate specificities, the degradation process can be affected by the chemical structure of the polymer. The chemically synthesized polymers are recognized as lipid and cutin substrate analogs and use lipase and cutinase, respectively, to hydrolyze ester bonds. In contrast, lipase, and cutinase cannot hydrolyze PHAs, including PHBH, but poly(3HB) depolymerase can recognize them as matched substrates [6, 13]. Moreover, the enzymatic hydrolysis of each polymer generates a different monomer structure, thereby differentiating the downstream metabolic pathways. Such reacting pathways and resource structure variety provide unique microbial habitats depending on the polymer materials. Previous studies have described unique microbiomes on plastics using DNA sequencing approaches, including amplicon and shotgun metagenomic sequencing [14–18]. Such studies usually compare conventional plastics with environmental water or a single biodegradable polymer with other conventional polymers. Few studies have compared and verified the differences in microbial communities among various biodegradable polymers.

To understand the degradation processes, capturing the temporal changes in microbial communities would also be required. Plastics degradation comprises several steps: (1) pioneer microbes adhere on the polymer surface stochastically, (2) microbes capable of degrading the polymer are selected on the surface community, then (3) these degraders are overtaken by subsequent consumers [14, 19]. Such a successional pattern would manifest as microbial compositional and functional differences.

Several standardized methods for evaluating biodegradable polymer degradation have been presented by ISOs (e.g., ISO 18830 for seawater, and ISO 17556 for soils). However, they all have problems with prolonged degradation experiments, requiring a maximum testing period of 2 years. Therefore, a prompt evaluation for polymer degradation is necessary to collect large datasets to cover whole degradation processes and determine a common principle for the community assembly.

In this study, we set up prompt evaluation systems for polymer degradation *in vivo* by concentrating the microbial biomass and adding nutrients for its growth. This prompt evaluation system allows us to observe wide-range successional stages on plastispheres within a short period. We investigated both the microbiome and metabolome related to the degradation of six biodegradable polymers (PHBH with 10% 3HH, PHBH with 6% 3HH, PCL, PBSA, PBS, and PBAT; Fig. S1, Table S1). This rapid evaluation approach made possible five repetitive tests within a short time period, resulting in a total of 418 microbiomes (16 S rRNA amplicon sequences) and 125 metabolomes (nuclear magnetic resonance [NMR] spectroscopy). The alignment information of the amplicon was also used for PICRUSt2 analysis to predict the microbiome functions. To the best of our knowledge, this is the first attempt to integrate such large DNA and NMR spectroscopy datasets to characterize different biodegradable polymer-related degradation processes.

## Materials and methods

### Incubation experiments

We performed incubation experiments at five different times (Table 1). Brackish water containing bottom sediment was sampled from a tidal flat at the mouth of the Tsurumi River (35°5ʹ N, 139°7ʹ E) five times from September 2020 to April 2021 (Table 1). To accelerate microbial degradation *in vivo*, we collected microbes on 0.2-µm Ominipore® membranes after pre-filtrating the collected water through 10-µm Ominipore® membranes, which was concentrated in the environmental water (Table 1 shows the concentration rate for each test). For time-series sampling, we prepared containers for the number of sampling points, in which the microbe-concentrated water was dispensed. Subsequently, polymer sheets (PHBH with 10% or 6% 3HH, PCL, PBSA, PBS, and PBAT; product information in Table S1 and Fig. S1) were added into the bottles. These polymer sheets were prepared by melting the polymer products with heat, then pressing (Table S1). After adding nutrients for microbial growth (0.5 g/L of NH_4_Cl and 0.1 g/L of KH_2_PO_4_, based on the international standard of ASTM D6691-17), the bottles of water were shaken at 160 rpm in an incubator at 25 °C. This study integrated five different experiments into one paper, and each experiment had a different experimental design (Table 1), where the incubation started with different water samples, different polymer materials, different sheet sizes, and different incubated water volumes. The sampling intervals were different among the five tests; we sampled the bottles intensively at the early decomposition stage in Tests D and E. Since we integrated five different tests in this study, the experimental settings were largely different. Note that we do not focus further on the difference among the five tests since a lot of experimental settings vary. Instead, we focus on the effects of polymer niche and succession (i.e., polymer type and incubation day) by statistically separating them with the “test” effect. We picked up the bottles at each sampling point and collected the polymer sheets (Tests A–E) and the remaining incubated water (Tests A–C). We did not collect the incubated water in Tests D and E because the incubations were performed with insufficient water volumes for NMR analysis. To assess the microbiomes of the biofilms and polymer surfaces separately, the polymer sheets were vortexed, then centrifuged at 15 000 rpm, and we defined the precipitates after the centrifugation as “biofilm” components and those remaining on the polymer as “surface” components. We separated the biofilms and polymer surfaces, except for in Test D. The degraded polymer (surface sample), biofilm, and incubated water were stored at −30 °C, then lyophilized for subsequent analysis.

**Table 1:** Information for each experiment.

Test	Start date	Material	Polymer shape	Water volume (mL)	Concentration ratio	Sampling points	Biofilm-Surface Separation	Weight loss BOD measurement	Microbiome	Metabolome
Test.A	Sep.3. 2020	PHBH(10%-3HH), PCL, PBSA, PBS, PBAT	Rectangle 1.0 × 6.0 × 0.05 cm^3^	40	×10	2 d, 4 d, 7 d, 11 d, 14 d, 21 d, 28 d, 35 d, 42 d, 49 d, 56 d	**○**	**○**	**○**	**○**
Test.B	Oct.1. 2020	PHBH(10%-3HH), PCL, PBSA	Rectangle 1.0 × 6.0 × 0.02 cm^3^	40	×10	4 d, 7 d, 14 d, 21 d, 28 d, 35 d, 42 d	**○**	**○**	**○**	**○**
Test.C	Nov.16. 2020	PHBH(10%-3HH), PHBH(6%-3HH), PCL, PBSA, PBAT	Rectangle 0.5 × 6.0 × 0.02 cm^3^	40	×8	1 d, 2 d, 3 d, 4 d, 7 d, 10 d, 14 d, 21 d, 28 d, 35 d, 42 d, 49 d, 56 d	**○**	**○**	**○**	**○**
Test.D	Feb.2. 2021	PHBH(10%-3HH), PHBH(6%-3HH), PCL, PBSA	Circular 0.25^2^π × 0.02 cm^3^	1	×20	4 h, 8 h, 16 h, 1 d, 2 d, 3 d, 4 d, 5 d, 6 d, 7 d, 10 d, 14 d	**×**	**×**	**○**	**×**
Test.E	Apr.2. 2021	PHBH(10%-3HH), PHBH(6%-3HH), PCL, PBSA	Rectangle 0.5 × 2.0 × 0.02 cm^3^	8	×10	6 h, 12 h, 18 h, 24 h, 30 h, 36 h, 42 h, 48 h, 60 h, 3 d, 4 d, 5 d, 6 d, 7 d, 8 d, 11 d, 14 d, 21 d	**○**	**×**	**○**	**×**

We tracked the polymer degradation processes by measuring the weight loss rate and consumption of biochemical oxygen demand (BOD) for Tests A–C. The weight loss rate was evaluated as the ratio of the weight loss to the original weight. For the BOD measurement, we prepared another incubation system and kept track of BOD consumption every day using an OxiTop® system. BOD consumption was calculated by subtracting BOD consumption in blank water (without polymer materials) from BOD consumption in water containing the polymer materials.

### 16 S rRNA amplicon sequencing

To determine the microbiome of each biofilm/surface sample, we performed amplicon analysis of the 16 S rRNA genes using a next-generation sequencer. DNA was collected from the biofilm and polymer surface samples by ethanol precipitation after phenol chloroform extraction, then the DNA was PCR-amplified using forward and reverse primers (Eurofins Genomics K.K., Tokyo, Japan), dNTP, and Ex Taq (Takara Bio Inc., Shiga, Japan). The PCR products were purified using AMpure (Beckman Coulter, Inc., Brea, USA), then redissolved in pure water. The concentration of the purified DNA was determined using a Qubit Fluorometer (Thermo Fisher Scientific, Waltham, USA), then the purified DNA was diluted to the same concentration and mixed into one library. The pooled library and PhiX Control v.3 (Illumina, Inc., San Diego, USA) were denatured with NaOH, then diluted to a final concentration of 3.5 pM with HT1 hybridization buffer and spiked with denatured PhiX solution. The final solution was loaded into MiSeq v2 Reagent Kits (Illumina, Inc., San Diego, USA) and analyzed using MiSeq (Illumina, Inc., San Diego, USA).

The sequence data in FASTQ format were processed using QIIME2 [20]. The read sequences were demultiplexed, then denoised to create amplicon sequence variant (ASV) clusters using DADA2. Each ASV was assigned using the Silva database to provide taxonomic information. Samples with more than 7 313 reads were used for the following analyses. The information on the ASVs was also used for the pipeline of PICRUSt2 [21] and annotated using KEGG Orthology (KO) to predict the potential functional gene profiles of the whole microbiome.

### Nuclear magnetic resonance (NMR) analysis

We determined the metabolites in the incubated water using NMR. The lyophilized samples were extracted with potassium phosphate (KPi) buffer solution in D_2_O, containing 1 mmol/L sodium 2,2-dimethyl-2-silapentane-5-sulfonate (DSS) at 65 °C for 15 min under shaking (1 400 rpm). After centrifugation at 14 000 rpm for 5 min, the supernatants were transferred into NMR tubes.

All samples were measured using an AVANCE II 700 MHz NMR spectrometer (Bruker BioSpin, Rheinstetten, Germany). We used a pulse program of 2D *J*-resolved (2D*J*-res) to focus on small-molecular-weight decomposed products. For metabolite annotation, we also measured the ^1^H–^13^C heteronuclear single quantum coherence of several samples. The row spectra of 2D*J*-res were processed with rNMR software [22]: we defined the region of interest (ROI) of 127 peaks, then calculated each peak intensity. For the following statistical analysis, the ROI intensities were normalized by the probabilistic quotient normalization method [23]. We used SpinAssign [24] and SpinCouple [25] to narrow down the candidate materials for each ROI, then annotated them by superimposing the standard material spectra. We annotated 71 of the 127 peaks (Table S2).

### Statistical analysis and network visualization

All statistical analyses and visualizations were performed with R software v.4.0.4. We performed non-metric multidimensional scaling (nMDS) for the microbiomes at ASV level, predicted KOs, and metabolomes using the ‘vegan’ package. The nMDS was based on the distance matrix of the Bray–Curtis index with the smallest number of axes for which the stress value was less than 0.2. Four-way PERMANOVA was applied to confirm the significance and the effects size of four factors (tests, polymer material, incubation time, and biofilm/surface). Microbiome/KO/metabolome differences among the polymer materials were evaluated by non-parametric multiple comparisons (Steel–Dwass method) for the Bray–Curtis dissimilarity index grouped by each test and polymer pair. For the microbiome dataset, the above statistical test was also applied after rarefaction treatment with a minimum read number of 7 313.

Our central interest in this study was to verify the effects of polymer materials (i.e., niche) and incubation time (i.e., succession) on the microbiome. To integrally analyze the whole dataset with different experimental designs and focus on these effects, the axis values of nMDS were regressed with a generalized linear mix model (GLMM) [26] based on the analytical framework of the Bayesian hierarchical modeling using the ‘brms’ package [27]. The models explain the target axis value by incubation time, polymer material, their interactions, and random effect for intercept of tests and biofilm/surface. The model we constructed was as follows:

$$r_k \sim Normal\left( {0,\sigma _\gamma ^2} \right)$$

$$g\left( {\mu _i} \right) = \beta _0 + {\sum} {\beta _jx_{ji} + r_k}$$

$$y_i \sim Normal\left( {\mu _i,\sigma ^2} \right)$$

where the random effect of the intercept *r*_*k*_ followed a normal distribution with a mean of 0 and a variance of $\sigma _\gamma ^2$; *β*_0_ is an intercept; *β*_*j*_ is a slope of fixed effects of *x*_*ji*_, including incubation time, polymer material, and their interactions, and a target variable follows a normal distribution with a mean of *μ*_*i*_ and a variance of σ^2^. We performed a parameter estimation using the Markov chain Monte Carlo method. The Bayesian inference started with uninformative priors for 3 000 iterations in four chains. In each chain, the first 2 000 iterations were used for warm-up, and the remaining 1 000 iterations were used for parameter estimation.

We constructed a co-occurrence network for the multi-omics dataset using the Apriori algorithm [28, 29] on the “arules” package with the matrix of experimental parameters (test, incubation time, polymer material, and biofilm/surface), metabolites, and microbes at the genus level. For metabolite and microbe data, the top 25% values of each parameter were converted to 1 and the rest were converted to 0. The categorical data of the experimental conditions were also binarized to 1 and 0. The incubation times were grouped into three categories: early (<4 days), middle (4–14 days), late (<14 days), which were also binarized. We determined the association rules with the threshold value of 0.0625 (0.25 × 0.25) for “support” value and 0.25 for “confidence” value as these values represent the probability of random occurrence in the case that the top 25% were binarized [28]. To visualize more strongly related links, we selected the links between polymer material nodes and multi-omics parameters greater than the median of the lift values. Network visualization was performed with the “tidygraph” and “ggraph” packages.

## Results

In this study, five prompt degradation experiments for five biodegradable polymers {PHBH (with 10% and 6% 3HH), PCL, PBSA, PBS, and PBAT} were conducted with condensed estuary water. Microbiome and metabolome data for each time-series point were obtained without separating surface and biofilm for test D and with surface and biofilm separated for the other tests. Detailed information on the experiments were summarized in Table 1.

### Degradation rates

**Fig. 1:**
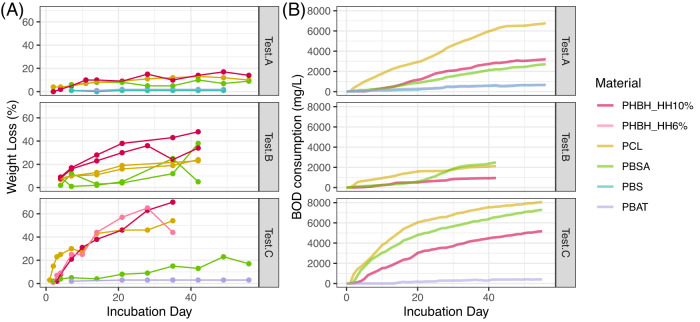
Degradation processes during the incubation in Tests A–C. (**A**) Temporal changes of Weight loss ratio (%) and (**B**) those of accumulated BOD consumption (mg/L). The point colors show different polymer types (Red: PHBH_10%, Pink: PHBH_6%, Yellow: PCL, Green: PBSA, Blue: PBS, and Purple: PBAT).

We tracked the degradation process by measuring the weight loss rate and BOD consumption in tests A–C. Both methods demonstrated that PHBH, PCL, and PBSA showed high decomposability, while the decomposition of PBS and PBAT were almost negligible (Fig. 1). In the tests A–C, the weight loss reached up to 70% for PHBH, 54% for PCL, 38% for PBSA, 1% for PBS, and 3% for PBAT. The BOD consumption reached up to 4045 mg/L for PHBH, 7078 mg/L for PCL, 7096 mg/L for PBSA, 332 mg/L for PBS, and 389 mg/L for PBAT.

### Microbiome analysis

The microbiomes of the 16 S rRNA amplicon sequences were obtained for all five tests, producing a total of 418 data points for statistical analysis (101 for PHBH with 10% HH, 68 for PHBH with 6% HH, 101 for PCL, 101 for PBSA, 14 for PBS, and 33 for PBAT). Microbiome compositions were shown at each taxonomic level (Figs. S2–S5). Almost all sequences were derived from bacterial sequences (89.7–100%, median of 100%), while those of archaea were negligible. Proteobacteria were the predominant phylum (9.8%–99.8%, median of 87.5%, Fig. S2), which was dominated by two classes: *Gammaproteobacteria* (1.8–98.6%, median of 56.9%, Fig. S3) and *Alphaproteobacteria* (0.5–96.4%, median of 21.5%, Fig. S3). It is noteworthy that in Tests D and E, the class Campylobacteria also dominated at the early stage of PBSA decomposition (0–14 days) (Fig. S3). At the family level, Comamonadaceae (0%–94.4%, median of 8.8%, Fig. S5) and Pseudomonadaceae (0–90.2%, median of 6.7%, Fig. S5) were typically observed at larger proportions regardless of the polymer material.

**Fig. 2:**
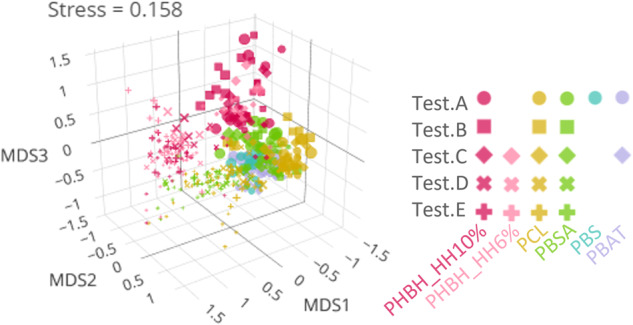
Nonmetric multidimensional scaling (nMDS) score plot of amplicon sequence variant (ASV)-level microbiome. The smallest axis number with stress values less than 0.2 was selected, that is three dimensions. The point colors show different polymer types (Red: PHBH_10%, Pink: PHBH_6%, Yellow: PCL, Green: PBSA, Blue: PBS, and Purple: PBAT), the point shapes show different test series (Circle: Test.A, Square: Test.B, Triangle: Test.C, Cross.1: Test.D, and Cross.2: Test.E), and the point size show incubation time.

The effects of experimental factors on the β-diversity of the ASV-level microbiome were calculated by PERMANOVA and then visualized by nMDS. Four-way PERMANOVA demonstrated that the microbial composition varied with four main factors: test (R^2^ = 0.158, *p* < 0.05), polymer material (R^2^ = 0.100, *p* < 0.05), incubation time (R^2^ = 0.028, *p* < 0.05), biofilm/surface (R^2^ = 0.007, *p* < 0.05), and some of their interactions (Table S3). The nMDS plot clearly showed that the most distinctive difference appeared between PHBH and other polymers (Fig. 2). The difference between each of the polymer materials were statistically analyzed by comparing the Bray–Curtis index between each polymer pair in each test (Steel–Dwass test, *p* < 0.05, Fig. S7). The Bray–Curtis index was lowest for pairs within the same polymer material in all cases. There was no difference in Bray–Curtis index between PHBH 10% HH and PHBH 6% HH. For all polymer materials except PHBH, the Bray–Curtis indices for PHBH samples were higher than those for non-PHBH samples. For PBS and PBAT, the Bray–Curtis indices were lower for pairs with PBSA rather than for pairs with PCL. These analyses were also performed for the ASV table after rarefaction treatment, confirming that there was no difference in the results between the treated and untreated datasets (Figs. S9, S10, Table S3).

**Fig. 3:**
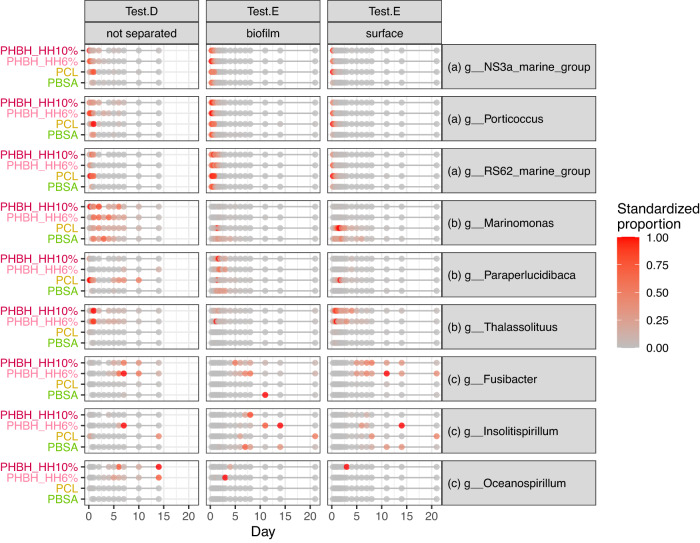
Time-series shift of microbial relative abundance for selected genera in Tests D and E. The relative abundance of each genus of each test was standardized to the maximum value of 1, and the standardized relative abundance was shown with a color gradient from gray to red. Based on the successional pattern, we set three groups for the genera. **a** early (Group.I): microbes whose abundance was the largest at the beginning and rapidly decreased (**b**) intermediate (Group.II): microbes which increased their abundance in parallel with the decline of Group.I and then decreased, and (**c**) late (Group.III): microbes which increased their abundance after the decline of Group.II.

In Tests D and E with a higher resolution for time series, we identified several genera which gradually change their relative abundance with time. The selected genera were shown in Figs. 3 and S14; some genera showed initial dominance at the beginning to immediately decrease with time (e.g., *NS3a_marine_group*, *Porticocccus*, and *RS62_marine_group*), convex shifts with higher abundance in the middle term (e.g., *Marinomonas*, *Paraperlucidibaca*, and *Thalassolituus*), and dominance in the late stage (e.g, *Fusibacter*, *Insolitispirillum*, and *Oceanospirillum*).

### Metabolome analysis

**Fig. 4:**
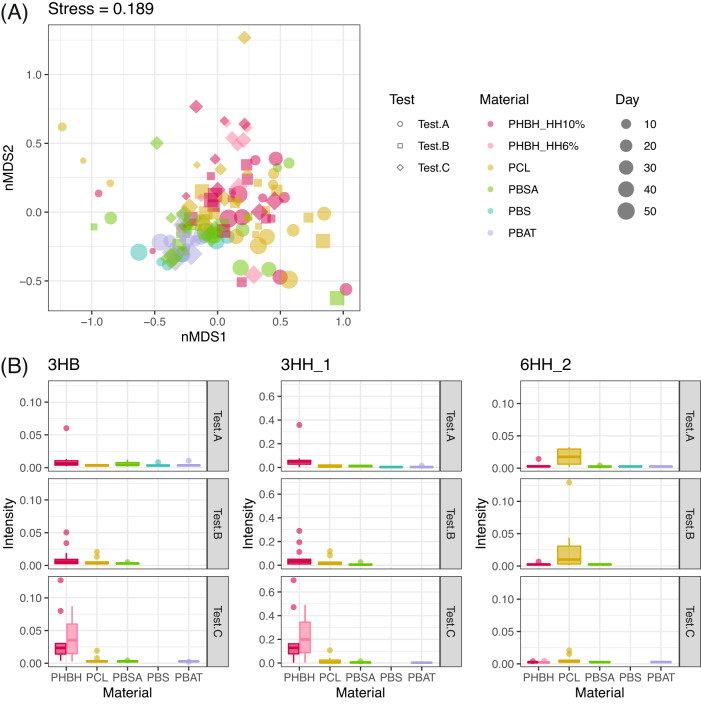
Metabolome analysis in the incubated water. **A** nMDS score plot for the signal intensities of NMR. The point colors show different polymer types (Red: PHBH_10%, Pink: PHBH_6%, Yellow: PCL, Green: PBSA, Blue: PBS, and Purple: PBAT), the point shapes show different test series (Circle: Test.A, Square: Test.B, and Rhombus: Test.C). The point size show incubation time. **B** The relative signal intensities of the selected metabolites (3HB, 3HH, and 6HH) for each polymer type and each test. The y-axis shows the relative peak intensity of the target signal when the integral of all NMR signals was set to 1.

We obtained 125 metabolome profiles of the incubated water in tests A–C using NMR. We defined a total of 127 peaks detected in 2D*J*-res spectra as ROI, for which the peak intensity was determined by rNMR [22]. We annotated 71 ROIs, including the monomer structures of the six polymer materials; namely, 6-hydroxyhexanoate (6HH), 3-hydroxybutyrate (3HB), 3-hydroxyhexanoate (3HH), 1,4-butanediol (BDO), succinate (SuA), and adipate were detected, while the peaks of terephthalate, a PBAT monomer, were not found. The differences in whole metabolic compositions were visualized by nMDS (Fig. 4). The results of the three-way PERMANOVA indicated that the metabolic profiles in the incubated water were different among the tests (R^2^ = 0.065, *p* < 0.05), polymer materials (R^2^ = 0.168, *p* < 0.05), incubation times (R^2^ = 0.028, *p* < 0.05), and their interactions (Table S3). The difference between the polymer types was ascribed to the monomer signals; a higher proportion of 6HH was detected for PCL, while a higher proportion of 3HB and 3HH were detected for PHBH (Fig. 4).

### Co-occurrence network

**Fig. 5:**
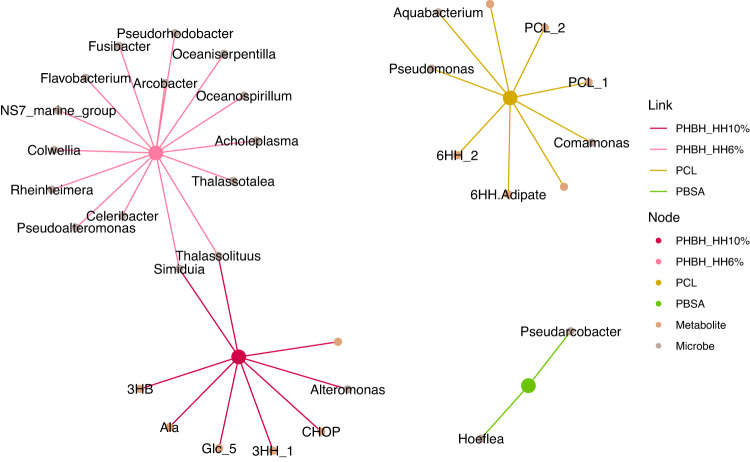
A cooccurrence network for the multi-omics (the proportion of each genus-level microbe, the proportion of each NMR signal, and experimental factors) dataset based on the Apriori algorithm. For the visualization, only the links with “polymer material” nodes with lift values greater than the median of all lift values were extracted. Edge colors show the links with different polymer materials, (Red: PHBH_10%, Pink: PHBH_6%, Yellow: PCL, Green: PBSA). The PBS and PBAT nodes were not extracted because of the small number of the data. The nodes with no label were non-annotated NMR signal. The annotated NMR signals are degraded polymer structures (3HB and 3HH for PHBH, PCL and 6HH for PCL) and low molecular metabolites (Ala: alanine, Glc: glucose, CHOP: Phosphocholine, and adipate). The other labeled nodes are microbial genera.

We constructed a co-occurrence network based on the Apriori algorithm, which revealed polymer-specific microbes and metabolites (Fig. 5). The PHBH node had links with its monomers (i.e., 3HH and 3HB), some metabolites (phosphocholine: CHOP, alanine: Ala, glucose: Glc), and microbes (e.g., *Thalassolituus*, *Simiduia*, *Alteromonas*, *Thalassotalea*, *Rheinheimera*, *Oceaniserpentilla*, *Flavobacterium*, etc.). The network groups of PHBH 10% HH and PHBH 6% HH appeared to be separated even though there was no difference in microbiome between them according to nMDS. This was thought to be because the number of test trials differed (5 tests for 10% HH, 3 tests for 6% HH), and the Apriori algorithm observes co-occurrence with both 10% HH and 6% HH only for microbes that are consistently dominant in all tests. The PCL node had links with its structural components (6HH and lower-molecular-weight PCL) and microbes (e.g., *Pseudomonas*, *Aquabacterium*, and *Comamonas*). PBSA had no links with their monomer, while it was associated with certain microbes assigned to the genera *Pseudarcobacter* and *Hoeflea*.

### Function prediction based on 16 S rRNA gene abundances

**Fig. 6:**
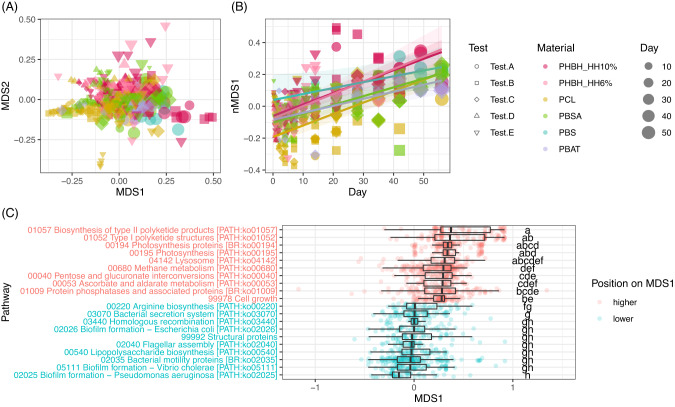
Multivariate analyses for the predicted KEGG orthologies (KOs). (**A**) nMDS score plot for the KOs. (**B**) Regression of Bayesian hierarchical model based on generalized liner mix model (GLMM) for nMDS axis.1 with incubation time, polymer material, their interactions, and random effects of test and surface/biofilm. For the figures (**A**) and (**B**), the point colors show different polymer types (Red: PHBH_10%, Pink: PHBH_6%, Yellow: PCL, Green: PBSA, Blue: PBS, and Purple: PBAT), the point shapes show different test series (Circle: Test.A, Square: Test.B, Rhombus: Test.C, Triangle.1: Test.D, and Triangle.2: Test.E), and the point size show incubation time. (**C**) Boxplots summarizing the score values of each KO on the nMDS-1 axis in the pathway level. Only the top 10 highest (red color) and lowest (blue color) pathways are shown. The score values for each pathway were compared by a non-parametric multiple comparison (Steel–Dwass method), with significant differences shown by different alphabet.

To extend the functional insights from the 16 S amplicon sequencing, we predicted the metagenome functions using PICRUSt2 [21], in which the sequence data were converted to the predicted functional abundance based on the KEGG database. A total of 10 543 KOs were annotated for 418 samples, of which the dimensions were reduced into two by nMDS (Fig. 6A). The whole profiles of the predicted KOs were different among the tests (R^2^ = 0.150, *p* < 0.05), polymer materials (R^2^ = 0.089, *p* < 0.05), incubation times (R^2^ = 0.83, *p* < 0.05), biofilms/surfaces (R^2^ = 0.013, *p* < 0.05), and some of their interactions (four-way PERMANOVA, Table S3). Importantly, the values of nMDS axis-1 were positively associated with the incubation time (Fig. 6B) for almost all time-series sets (Fig. S12). The scores of each KO on nMDS axis.1 were categorized into 533 pathway groups based on the KEGG database, and the ten pathways with the highest and lowest scores on the nMDS axis.1 were extracted (Fig. 6C). The lower scores of early successions are likely to be related to biofilm formation (KO02025, KO05111, KO02026, KO03070) and microbial motility (KO02035, KO02040). The higher scores of late successions are likely to be related to various functions, for example, the metabolism of polyketide (KO01057), methane (KO00680), ascorbate (KO00053), photosynthesis (KO00194), and cell growth (KO99978).

## Discussion

In this study, we obtained 418 microbiomes, and 125 metabolomes for five different polymers (PHBH (10%_HH and 6%_HH), PCL, PBSA, PBS, and PBAT) in a total of five experiments. To the best of our knowledge, this is the first study reporting the successional patterns on different biodegradable polymers based on such a large dataset. This comprehensiveness provides robust patterns and interpretation concerning biodegradable polymer degradation.

### Degradation process

We measured degradation rate by weight loss and BOD consumption in Tests A–C. Both weight loss and BOD consumption displayed a similar pattern; PHBH, PCL, and PBSA were decomposed relatively quickly, while the decomposition of PBS and PBAT were almost negligible (Fig. 1). These results are consistent with those of previous studies, which have demonstrated almost no biodegradability for PBS and PBAT in seawater [30, 31]. This difference could be because the microbial degraders of PBS and PBAT are sparse, while those of PHAs, including PHBH, and PCL are ubiquitous in marine ecosystems [4]. Although the structure of PBSA resembles those of PBS and PBAT, PBSA shows higher decomposability because its low crystallinity makes it more susceptible to enzymatic reactions [32, 33].

### Niche differences of microbes among polymer materials

Interestingly, the microbial community compositions varied significantly depending on the polymer type (four-way PERMANOVA, *p* < 0.05). The most noticeable discrimination was observed between PHBH and the other polymers (Figs. 2, S7), in line with the opposition of biologically and chemically synthesized polymers. These differences in the microbiome between PHBH and other polymers were clearly observed when the five tests were combined (Fig. 2), indicating that the microbiome composition converged for each polymer type to some extent regardless of the initial microbiome and experimental conditions. Such a distinctive microbial community structure on PHAs, to which PHBH belongs, has also been reported in a previous study [18]. This gap in community structure is likely to be attributable to the microbial capability to use polymer materials as resources. PHAs, including PHBH, are ubiquitous in nature and are synthesized by specific microbes for intracellular energy storage when they grow in carbon-rich but nutrient-deficient environments [6]. The degradation of PHAs starts with the extracellular enzymatic cleavage of ester bonds. For these naturally occurring PHAs, specific microbes have developed a specialized degrading enzyme: poly(3HB) depolymerase [6, 34, 35]. In contrast, chemically synthesized polymers, including PCL, PBSA, PBS, and PBAT, do not exist in nature and cannot be degraded by poly(3HB) depolymerases, only by lipase and cutinase as lipid or cutin analogs [6, 13]. Lipase and cutinase cannot hydrolyze most PHAs, and PHB depolymerase cannot hydrolyze other polymers [13, 34, 36]. Jaeger et al. exposed several PHA and PCL structures to nine and five PHA depolymerase and lipase types, respectively, and found that all poly(3HB) depolymerases could not degrade PCL and that lipase could not degrade PHAs, except for poly(4-HB) [13]. Such substrate specificity could have primarily defined the resource availability for the microbes, resulting in the distinctive community structure on PHBH. The second difference appeared between PCL and the other polymers (Figs. 2, S6, S7). Although these polymers were degraded by the same degrading enzymes, i.e., lipase, and cutinase, the discrimination was probably due to the resource structure differences between PCL and the other polymers. PCL consists of a single 6HH monomer, while the others are copolymers of 1,4-butanediol and dicarboxylic acids. Such structure differences would potentially differentiates the downstream metabolism, leading to the different microbiome compositions.

A co-occurrence network was constructed using the genus-level microbiome and metabolome datasets. Although the PBS and PBAT nodes exhibited no connection due to the small sample size, the network extracted polymer-specific microbes and metabolites (Fig. 4). Each polymer node was associated with its monomer (PHBH with 3HH and 3HB, PCL with 6HH and low-molecular weight PCL), except for PBSA (Fig. 5). Such node connections indicate that the monomers were released by polymer hydrolysis. The absence of PBSA-monomer edges is likely because the monomers (succinate and adipate) appeared not only on PBSA as hydrolyzed structures but also on other polymers as microbial metabolites since they are ubiquitous in biological metabolisms. The PHBH nodes were connected with many microbe nodes. Interestingly, most of the genera linked to PHBH on the network (9 of 16) belonged to the *Gammaproteobacteria* class, which is consistent with the fact that PHBH degraders were mainly found within *Gammaproteobacteria* [37]. Moreover, these extracted microbes included many genera that have been isolated as PHA-degraders in previous studies, for example, *Thalassolituus* [38], *Pseudoalteromonas* [38–41] *Alteromonas* [39], *Rheinheimera* [39], *Colwellia* [38], and *Aestuariibacter* [38]. This suggests that these genera possibly have the *phaZ* gene, which can produce PHA depolymerases, and therefore PHBH degradation ability. Other chemically synthesized polymers were also linked with specific microbial taxa (i.e., *Pseudomonas*, *Aquabacterium*, and *Comamonas* for PCL, *Pseudarcobacter* and *Hoeflea* for PBSA), even though they were degraded by the same types of hydrolases, possibly attributable to the differences in microbial metabolic pathways for each polymer.

In this study, we also expected that the microbiomes would be different between the surface and the biofilm; therefore, we separated them for the DNA analysis. The results of the four-way PERMANOVA showed that the microbiomes were significantly different between biofilm and surface, yet the contribution to whole β-diversity was remarkably small (Table S3, R^2^ = 0.007, *p* < 0.05). Conceptually, niche differentiations are likely to occur on the surfaces and biofilms of plastispheres due to the resource discrepancy; the surface microbes are likely to specialize in polymer degradation, while the biofilm microbes are likely to feed on extracellular polymeric substances or microbial detritus resulting from biofilm formation. Such a small contribution to biofilm/surface difference on microbiomes might suggest the lower importance of biofilm/surface location compared with the initial microbiome of different tests, polymer materials, and incubation times, or it is possible that the vortex separation used in this study was not sufficient to separate the surface and the biofilms.

### Microbial succession

In the nMDS for ASVs, we observed the microbiome shift over time (Figs. 2, S8). The Bayesian hierarchical model, in which the nMDS axes are explained by the polymer materials, the incubation times, and their interactions, as well as random effects, showed that the microbiome changed from a larger to smaller value on axis.1 and axis.2 during the incubations (Fig. S8). Such community shift patterns were observed regardless of the tests and polymer materials (Fig. S11), suggesting the homology of microbiome succession on the plastisphere.

In most cases, especially for PHBH and PCL, the dominant class, *Gammaproteobacteria*, was eventually replaced by *Alphaproteobacteria* (Fig. S13). Importantly, the intensive sampling in Tests D and E provided high resolution of the microbial succession, demonstrating that, in the initial transition, *Alphaproteobacteria* decreased rapidly toward the minimum proportion on days 2–3 and then recovered, whereas *Gammaproteobacteria* showed the opposite trend (Fig. S13). Such patterns can be interpreted as a process in which the initial microbiome, equivalent to the initial water environment, is replaced by microbes capable of degrading polymers (mainly *Gammaproteobacteria*), and then the proportion of biofilm components finally increased. *Alphaproteobacteria* are widespread in natural hydrospheres such as oceans and freshwater [42, 43], while the *Gammaproteobacteria* include a variety of plastic degraders. A recent comprehensive analysis summarized plastic degraders, including Eukaryota and Bacteria, on a phylogenetic tree, and demonstrated that 17.7% of the reported taxa were from *Gammaproteobacteria* and 3.0% were from *Alphaproteobacteria* [37]. Considering the above, the successional patterns of *Alphaproteobacteria* and *Gammaproteobacteria* may reflect the microbial functions to some extent, and the polymer degraders are likely to be found around 2–3 days in the early degradation stages in this case.

The dense time-series of the genera for Tests D and E also provided more detailed information on the initial microbial succession, indicating the contrasting successional patterns among genera. We summarized the transition trends for several interesting genera. Group I microbes, including *RS62* (*Comamonadaceae*), *Porticoccus* (Porticoccaceae), *NS3a* (Flavobacteriaceae), and other genera, had an initial peak of their proportion, which disappeared by Day 3 (Figs. 3, S14A). These groups may have been dominant in the water in the initial environment and then displaced by other dominant genera once they were exposed to the nutrient-rich environment of the biodegradable polymers. In addition, the abundance of Group I microbes exhibited little difference between the polymer types, indicating that these initial microbes adhered stochastically on the polymers regardless of their niche. Group II microbes, including *Thalassolituus* (Saccharospirillaceae), *Marinomonas* (Marinomonadaceae), *Paraperlucidibaca* (Moraxellaceae), and other genera, were almost absent at the very beginning, but increased in abundance to the maximum at around days 1–3, in parallel with the decline of Group I microbes, and finally decreased (Figs. 3, S14B). This group would include polymer degraders because the polymer materials are exposed intact in the environmental water in the initial state, and therefore the first step of the succession on polymers is likely to be determined by the bioavailability of the polymer materials. Group III microbes, including *Insolitispirillum* (Rhodospirillaceae), *Fusibacter* (Fusibacteraceae), *Oceanospirillum (*Oceanospirillaceae), and other genera, started to increase after the decline of Group II microbes (Figs. 3, S14C). This group is assumed to be the main component of biofilms since Group II microbes are polymer degraders and can live only on polymer surfaces. As microbial habitats accumulate on the biofilm layers, the ratio of Group II microbes relatively decreases. Despite only two tests of Test.D and Test.E for the dense sampling, it is interesting that the similar increase/decrease patterns above were observed. Previous studies have only showed the microbiome after several weeks to months in polymer degradation, which would miss capturing the detailed successional patterns for inferring the microbial roles. As observed in this study, the microbiome composition can change drastically in a few days, more dense monitoring for microbiome would be recommended to understand microbial turnover during polymer degradation.

Lastly, PICRUSt2 provided insights into the functional changes albeit predicted values rather than actual measurements. In the regression of the Bayesian hierarchical modeling which accounted for the differences in tests and surface/biofilm, nMDS axis.1 values were strongly positively related to incubation time (Figs. 6A, B; S12), suggesting that the variation on the axis.1 reflects time-series shift in the potential of microbial functions. To interpret the 10 543 KOs collectively, the axis.1 scores of each KO were integrated into 533 pathways to be represented in a boxplot. Interestingly, the pathways which occurred on lower axis.1, which indicates early successional stages, were mainly related to microbial motility (e.g., flagellar assembly) or biofilm formation (e.g., polysaccharide biosynthesis). This implies that free-swimming microbes with flagella were abundant in early successional stages, and the existence of microbes with biofilm-forming potential even in the early successional stages. This pattern may confirm a biofilm formation theory. First, free-swimming bacteria adhere stochastically [19] and some start to degrade the polymer materials or to produce extracellular polymeric substances, providing additional resources and habitats for microbes to grow exponentially and form a thick layer [14, 19, 44, 45]. However, since this scenario includes certain PICRUSt2-based estimations, direct metagenome-, and metatranscriptome-based measurements would be necessary to describe the functional shift during polymer degradation.

## Supplementary Information

Supplementary material

## Data Availability

All data and R source code are available via https://github.com/DYokoyama-shorea/Multiomics.plastisphere. An interactive plot of Fig. 2 is also available via https://github.com/DYokoyama-shorea/Multiomics.plastisphere/blob/main/Fig.2.html.
