# Supplementary material for: Large-scale omics dataset of polymer degradation provides robust interpretation for microbial niche and succession on different plastisphere
Source: ISME Commun. 2023 Jul 3;3:67. doi: 10.1038/s43705-023-00275-z (PMC10317964; doi:10.1038/s43705-023-00275-z)
Supplement: Supplementary file 1 — Supplementary material [file 43705_2023_275_MOESM1_ESM.docx]

**Supplementary material for:** Large-scale Omics Dataset of Polymer Degradation Provides Robust Interpretation for Microbial Niche and Succession on Different Plastisphere

**Authors:**

Daiki Yokoyama^1,2^, Ayari Takamura^1^, Yuuri Tsuboi^1^, Jun Kikuchi^1,2,3^

**Affiliation:**

1 RIKEN Center for Sustainable Resource Science, 1-7-22 Suehiro-cho, Tsurumi-ku, Yokohama, Kanagawa 230-0045, Japan

2 Graduate School of Medical Life Science, Yokohama City University, 1-7-29 Suehiro-cho, Tsurumi-ku, Yokohama, Kanagawa 230-0045, Japan

3 Graduate School of Bioagricultural Sciences, Nagoya University, 1 Furo-cho, Chikusa-ku, Nagoya, Aichi 464-0810, Japan

**Corresponding Author**

Jun Kikuchi

RIKEN Center for Sustainable Resource Science, 1-7-22 Suehiro-cho, Tsurumi-ku, Yokohama, Kanagawa 230-0045, Japan

jun.kikuchi@riken.jp

This PDF file includes:

Table S1 to S2

Figures S1 to S6

**Table S1** Information on polymers used in this study

| **Polymer** | **Product** | **Method to make sheet** | **Monomer** | **Synthetic method** | **Degrading enzymes** |
| --- | --- | --- | --- | --- | --- |
| PHBH  (10% 3HH) | Aonilex® X151A | Melted at 170°C for 7 min  Pressed at 20 MPa for 3min  Cooled at rt | 3HB  3HH | Biologically | 3HB depolymerase |
| PHBH  (6% 3HH) | Aonilex® X131A | Melted at 170°C for 7 min  Pressed at 20 MPa for 3min  Cooled at rt | 3HB  3HH | Biologically | 3HB depolymerase |
| PCL | PCL MW 80000  CAS: 24980-41-4 | Melted at 90°C for 7 min  Pressed at 20 MPa for 3min  Cooled at rt | 6HH | Chemically | Lipase  Cutinase |
| PBSA | BioPBS™  FD92PB | Melted at 110°C for 7 min  Pressed at 20 MPa for 3min  Cooled at rt | 1,4-butanediol  Succinate  Adipate | Chemically | Lipase  Cutinase |
| PBS | BioPBS™  FZ91PB | Melted at 130°C for 7 min  Pressed at 20 MPa for 3min  Cooled at rt | 1,4-butanediol  Succinate | Chemically | Lipase  Cutinase |
| PBAT | Ecoflex®  FCN 907 | Melted at 160°C for 7 min  Pressed at 20 MPa for 3min  Cooled at rt | 1,4-butanediol  Adipate  Terephthalate | Chemically | Lipase  Cutinase |

3HB: 3-Hydroxy butanoate, 3HH: 3-Hydroxy hexanoate, 6HH: 6-Hydroxy hexanoate

**Table S2** Annotation list of each ROI in 2D*J-res* spectra

| Chemical Shift  (ppm) | Annotation | Tag |
| --- | --- | --- |
| 0.88 |  |  |
| 0.90 | 3-Hydroxy hexanoate | 3HH_2 |
| 0.93 | Isoleucine | Ile_2 |
| 0.95 | Leucine | Leu_2 |
| 0.96 | Leucine | Leu_1 |
| 0.98 | Valine | Val_2 |
| 1.00 | Isoleucine | Ile_1 |
| 1.03 | Valine | Val_1 |
| 1.05 |  |  |
| 1.10 |  |  |
| 1.13 |  |  |
| 1.17 | Ethanol | EtOH_1 |
| 1.19 | 3-Hydroxy hexanoate | 3HH_1 |
| 1.21 |  |  |
| 1.26 | Isoleucine | Ile_3 |
| 1.32 | Lactate, Threonine | LA.Thr |
| 1.33 | 6-Hydroxy hexanoate | 6HH_2 |
| 1.35 |  |  |
| 1.39 |  |  |
| 1.47 | Alanine | Ala |
| 1.54 | Adipate | Adipate |
| 1.56 | 6-Hydroxy hexanoate, 1,4-butanediol | 6HH.BDO |
| 1.63 | 6-Hydroxy hexanoate | 6HH_3 |
| 1.66 | 6-Hydroxy hexanoate | 6HH_4 |
| 1.71 | Arginine, Lysine | Arg.Lys |
| 1.76 |  |  |
| 1.85 |  |  |
| 1.86 |  |  |
| 1.91 | Acetate | Ac |
| 2.00 | Proline | Pro |
| 2.03 | N-Acetyl group | NAct |
| 2.05 | Glutamate | Glu_3 |
| 2.13 | Methionine, Glutamate | Met.Glu |

**Table S2 continued**

| Chemical Shift  (ppm) | Annotation | Tag |
| --- | --- | --- |
| 2.18 | 6-Hydroxy hexanoate, Adipate | 6HH.Adipate |
| 2.22 |  |  |
| 2.27 | Valine | Val_3 |
| 2.29 | 3-Hydroxy butanoate | 3HB |
| 2.33 | Glutamate | Glu_1 |
| 2.34 | Glutamate | Glu_2 |
| 2.36 | Glutamate | Glu_4 |
| 2.39 | Succinate, 3-Hydroxy hexanoate, 6-Hydroxy hexanoate | SuA.3HH.6HH |
| 2.40 | Succinate | SuA |
| 2.46 |  |  |
| 2.54 | Beta-Alanine | bAla |
| 2.59 |  |  |
| 2.60 |  |  |
| 2.63 | Methionine | Met |
| 2.67 | Aspartate | Asp_2 |
| 2.70 |  |  |
| 2.73 | Dimethylamine | DMA |
| 2.74 | Sarcosine | Sarcosine |
| 2.78 |  |  |
| 2.80 | Aspartate | Asp_1 |
| 2.86 | Aspargine | Asn_1 |
| 2.88 | Trimehylamine | TMA |
| 2.94 | Aspargine | Asn_2 |
| 3.00 |  |  |
| 3.02 |  |  |
| 3.04 |  |  |
| 3.10 |  |  |
| 3.14 |  |  |
| 3.17 |  |  |
| 3.20 | Choline | CHO |
| 3.22 | Phospho choline | CHOP |
| 3.26 | Trimehylamin-N-oxide | TMAO |
| 3.28 |  |  |

**Table S2 continued**

| Chemical Shift  (ppm) | Annotation | Tag |
| --- | --- | --- |
| 3.34 |  |  |
| 3.38 |  |  |
| 3.39 | Glucose | Glc_3 |
| 3.40 | Glucose | Glc_10 |
| 3.46 | Glucose | Glc_4 |
| 3.48 | Glucose | Glc_5 |
| 3.53 | Glucose | Glc_2 |
| 3.55 | Glycerol,Glycine | Glycerol.Gly |
| 3.58 | Threonine | Thr_1 |
| 3.59 | decomposed PCL | PCL_1 |
| 3.64 | Glycerol, Ethanol, 1,4-butanediol | Glycerol.EtOH.BDO |
| 3.65 | Ethanol | EtOH_2 |
| 3.66 |  |  |
| 3.71 | Glucose | Glc_6 |
| 3.73 |  |  |
| 3.75 |  |  |
| 3.76 | Glucose | Glc_7 |
| 3.77 |  |  |
| 3.81 |  |  |
| 3.83 | Glucose | Glc_1 |
| 3.86 |  |  |
| 3.87 |  |  |
| 3.90 | Betaine, Uridine | Betaine.Uridine |
| 3.94 | Serine | Ser_1 |
| 3.95 |  |  |
| 3.96 | Serine | Ser_2 |
| 3.98 | Serine | Ser_3 |
| 3.99 | 3-Hydroxy hexanoate | 3HH_3 |
| 4.04 |  |  |
| 4.09 |  |  |
| 4.12 | decomposed PCL | PCL_2 |
| 4.24 | Threonine | Thr_2 |
| 4.31 |  |  |

**Table S2 continued**

| **Chemical Shift**  **(ppm)** | **Annotation** | **Tag** |
| --- | --- | --- |
| 4.41 |  |  |
| 4.64 | Glucose | Glc_8 |
| 4.94 |  |  |
| 5.19 | Trehalose | Trh |
| 5.23 | Glucose | Glc_9 |
| 5.61 |  |  |
| 5.79 |  |  |
| 5.94 |  |  |
| 5.97 |  |  |
| 6.02 |  |  |
| 6.11 |  |  |
| 6.45 |  |  |
| 6.89 | Tyrosine | Tyr |
| 7.31 | Phenylalanine | Phe_2 |
| 7.36 | Phenylalanine | Phe_3 |
| 7.42 | Phenylalanine | Phe_1 |
| 7.53 |  |  |
| 7.67 |  |  |
| 7.72 |  |  |
| 8.09 |  |  |
| 8.14 |  |  |
| 8.18 |  |  |
| 8.21 |  |  |
| 8.26 |  |  |
| 8.33 |  |  |
| 8.45 | Formate | FoA |
| 8.52 |  |  |
| 8.59 |  |  |

**Table S3.** Summary of PERMANOVA for microbiome data (raw ASV and rarefied ASV), metabolome data of NMR, and KEGG Orthologies (KOs) predicted by PICRUSt2. For microbiome and KOs, the analytical models were explained by four main effects (Test, Material, Incubation day, and biofilm/surface) and their interactions. For metabolome, the analytical models were explained by three main effects (Test, Material and Incubation day) and their interactions. The table shows the R^2^ value of each effect, with significant effects (p < 0.05) shown in bold.

| Dataset | Main Effects | | | | | | | | | | |  |  |  |  |  |
| --- | --- | --- | --- | --- | --- | --- | --- | --- | --- | --- | --- | --- | --- | --- | --- | --- |
|  | A  (Test) | | B  (Material) | | | C (Day) | | | D  (Biofilm/Surface) | | |  |  |  |  |  |
| ASV_raw | **0.158** | | **0.100** | | | **0.028** | | | **0.007** | | |  |  |  |  |  |
| ASV_rarefied | **0.157** | | **0.100** | | | **0.028** | | | **0.007** | | |  |  |  |  |  |
| NMR | **0.065** | | **0.168** | | | **0.028** | | | - | | |  |  |  |  |  |
| KOs | **0.150** | | **0.089** | | | **0.083** | | | **0.013** | | |  |  |  |  |  |
| Dataset | Interactions | | | | | | | | | | | | | | | Residual |
|  | A x B | A x C | | A x D | B x C | | B x D | C x D | | A x B x C | A x B x D | | A x C x D | B x C x D | A x B x C x D |  |
| ASV_raw | **0.087** | **0.050** | | **0.013** | **0.031** | | **0.010** | 0.001 | | **0.043** | 0.011 | | **0.008** | 0.003 | 0.007 | 0.442 |
| ASV_rarefied | **0.087** | **0.050** | | **0.013** | **0.031** | | **0.010** | 0.001 | | **0.043** | 0.011 | | **0.008** | 0.003 | 0.007 | 0.443 |
| NMR | **0.039** | **0.041** | | - | **0.041** | | - | - | | **0.046** | - | | - | - | - | 0.570 |
| KOs | **0.076** | **0.042** | | **0.017** | **0.026** | | 0.008 | 0.001 | | **0.039** | 0.011 | | **0.008** | 0.003 | 0.009 | 0.425 |

**
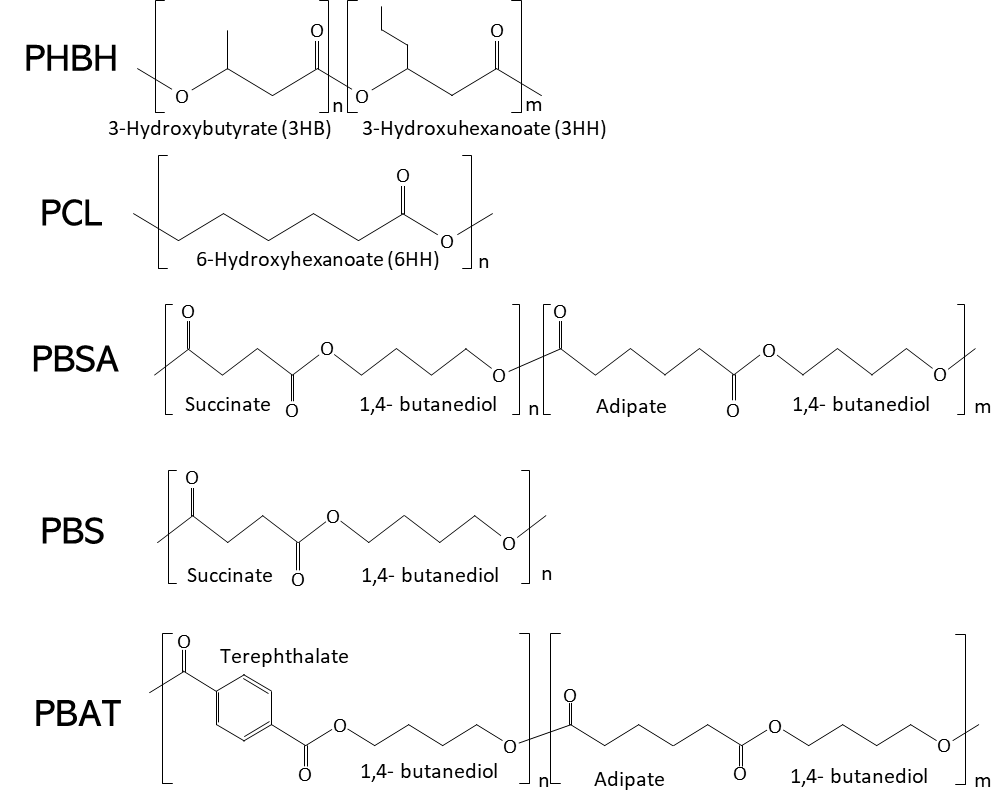
**

**Fig S1.** Chemical structure of polymer materials used in this study.


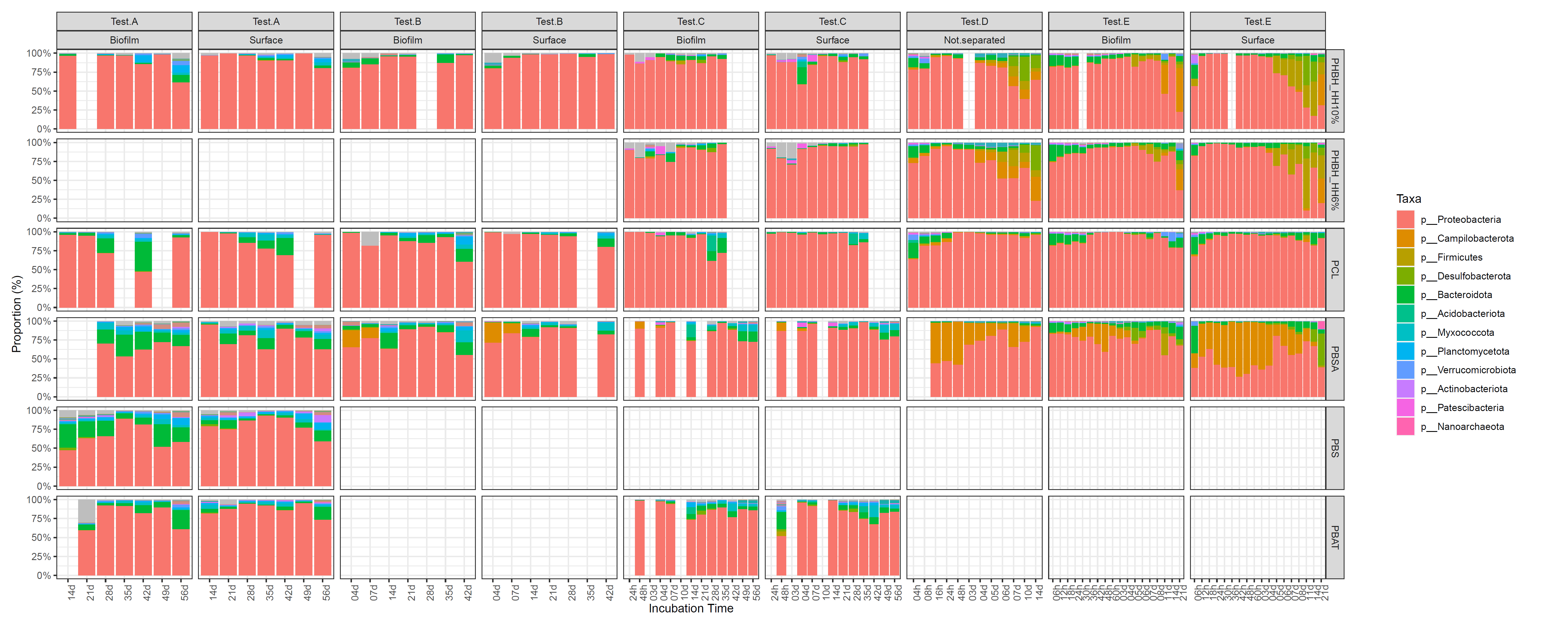
**Fig S2.** Microbiome composition at phylum level in all tests.

**Fig S3.** Microbiome composition at class level in all tests.


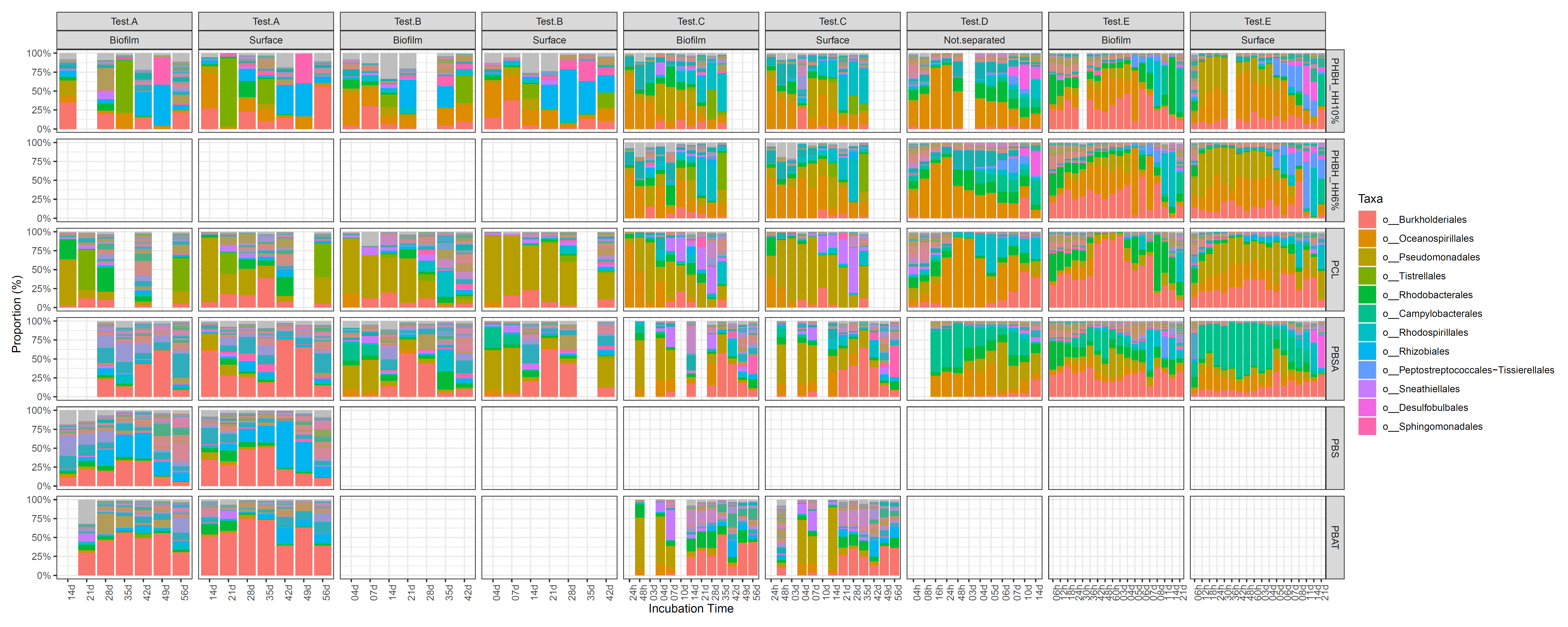


**Fig S4.** Microbiome composition at order level in all tests.


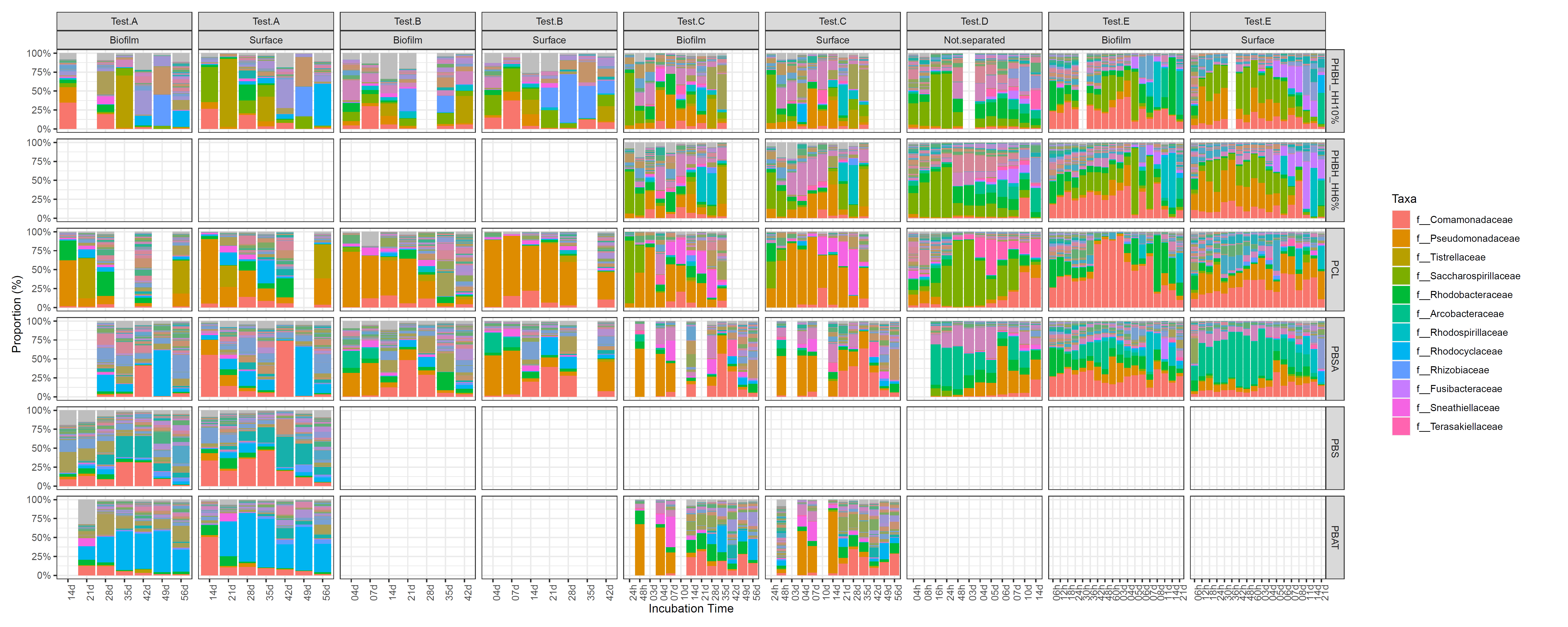


**Fig S5.** Microbiome composition at family level in all tests.

**
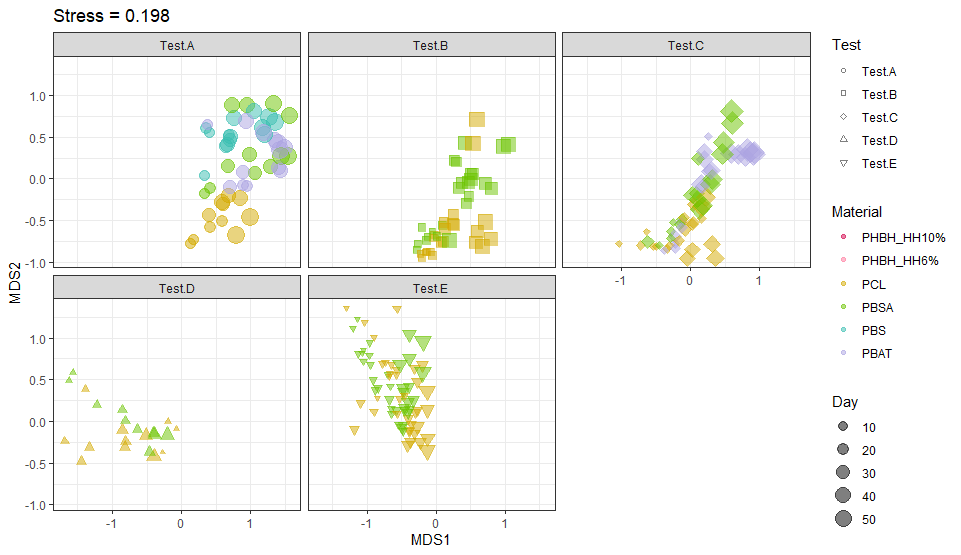
**

**Fig S6.** nMDS score plot for ASVs without 10%HH PHBH and 6%HH PHBH. The point colors show different polymer types (Yellow: PCL, Green: PBSA, Blue: PBS, and Purple: PBAT), the point shapes show different test series (Circle: Test.A, Square: Test.B, Rhombus: Test.C, Upepr triangle: Test.D, and Lower triangle: Test.E). The point size show incubation time.

**
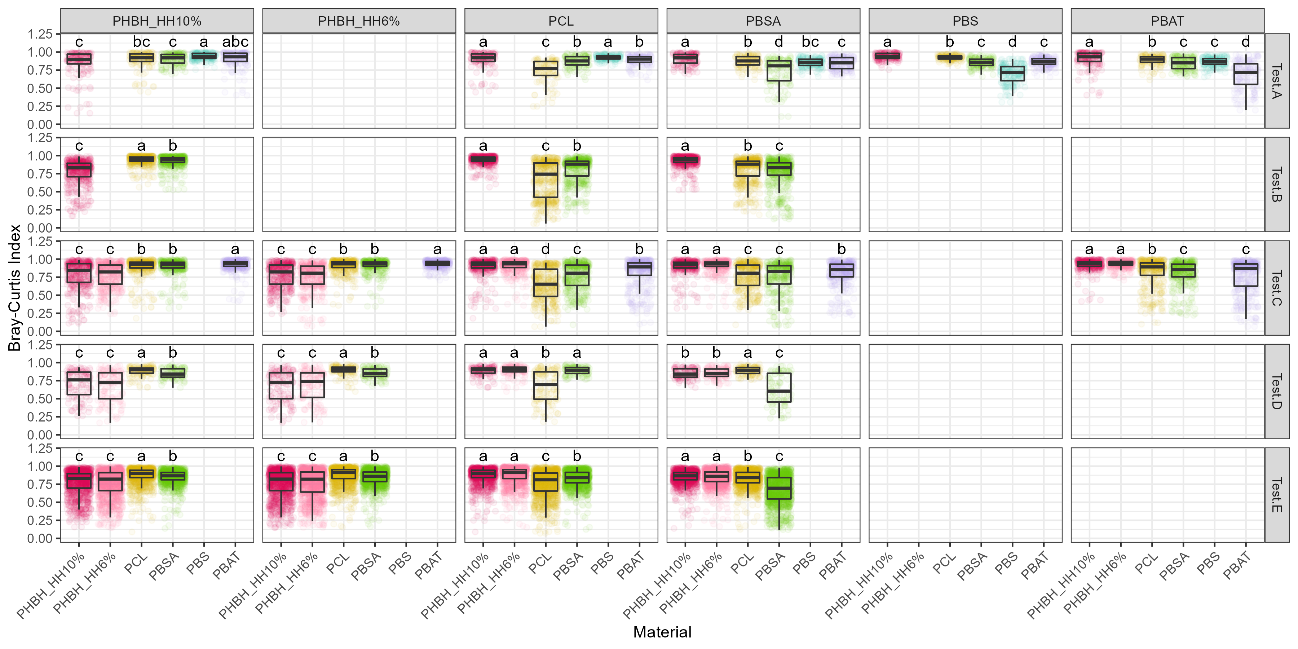
**

**Fig S7.** Bray-Curtis Index for each polymer pair in five tests. The Bray-Curtis index was calculated for each sample pair. Only the Bray-Curtis indices within the same tests were extracted and then visualized on the boxplot for each test and each polymer pair. Non-parametric multiple comparison (Steel-Dwass; p<0.05) was applied to compare Bray-Curtis indices among polymer pairs. The significant difference was described in different alphabet.

**Fig S8.** The results of GLMM for the nMDS axis of ASVs. The graphs show, from top to bottom, objective variables of axis.1, axis.2, and axis.3, and from left to right, the fixed effect of incubation day, polymer materials, and the interactions of incubation day and polymer materials.

**Fig S9.** An nMDS plot of the rarefied microbiome at ASV level. The point colors show different polymer types (Red: PHBH_10%, Pink: PHBH_6%, Yellow: PCL, Green: PBSA, Blue: PBS, and Purple: PBAT), the point shapes show different test series (Circle: Test.A, Square: Test.B, Rhombus: Test.C, Upepr triangle: Test.D, and Lower triangle: Test.E). The point size show incubation time.

**Fig. S10.** The comparison of Bray-Curtis index between polymer pairs and between raw/rarefied ASV tables. Non-parametric multiple comparison (Steel-Dwass; p<0.05) was applied to compare Bray-Curtis indices among polymer pairs. The significant difference was described in different alphabet.

**Fig S11.** The linear regressions of each nMDS axis value of ASVs with incubation day for each time-series.

 **Fig S12.** The regression of incubation day and nMDS axis.1 value of KOs for each time-series.


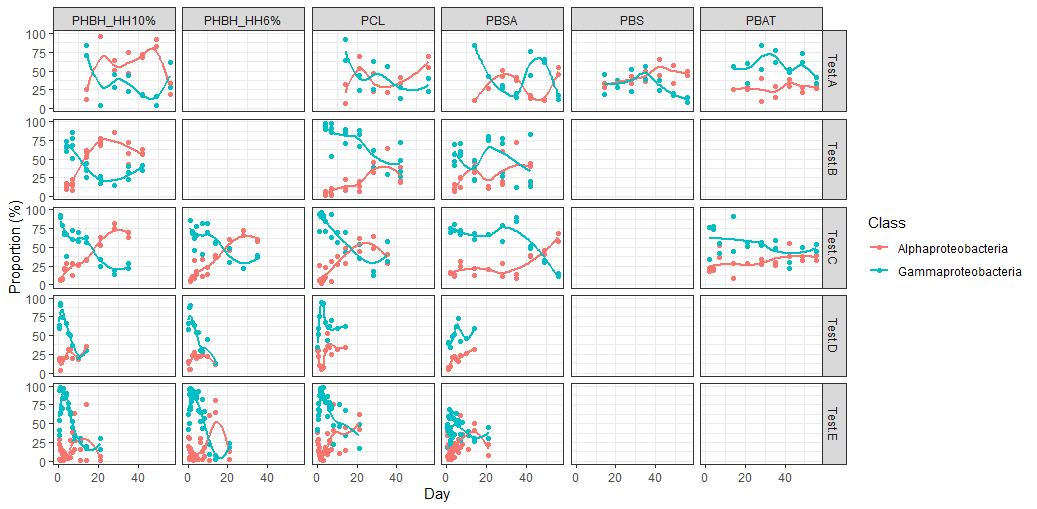


**Fig. S13** Relative abundance of two dominant classes of *Alphaproteobacteria* and *Gammaproteobacteria.*


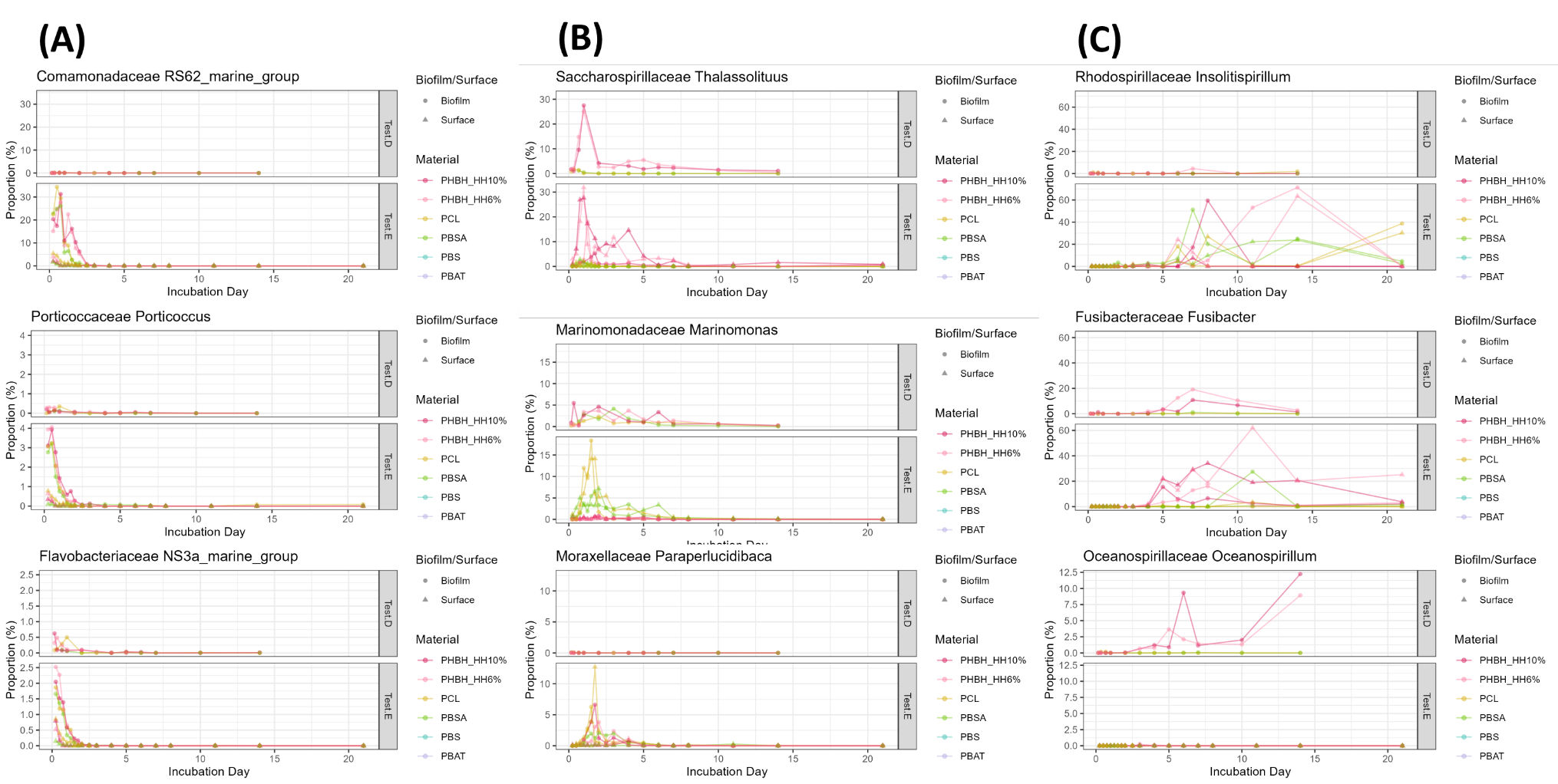


**Figure S14.** Examples of the relative abundance of selected genera. **(A)** Group.I; microbes whose abundance was the largest at the beginning and rapidly decreased, **(B)** Group.II: microbes which increased their abundance in parallel with the decline of Group.I and then decreased, and **(C)** Group.III microbes which increased their abundance after the decline of Group.II.
